# Phase-specific plasticity of synaptic structures in the somatosensory cortex of living mice during neuropathic pain

**DOI:** 10.1186/1744-8069-7-87

**Published:** 2011-11-09

**Authors:** Sun Kwang Kim, Go Kato, Tatsuya Ishikawa, Junichi Nabekura

**Affiliations:** 1 Division of Homeostatic Development, National Institute for Physiological Sciences, Okazaki, Japan; 2 Acupuncture & Meridian Science Research Center, Kyung Hee University, Seoul, Republic of Korea; 3 Department of Physiological Sciences, The Graduate School for Advanced Study, Hayama, Japan; 4 Core Research for Evolutional Science and Technology, Japan Science and Technology Agency, Saitama, Japan

## Abstract

**Background:**

Postsynaptic dendritic spines in the cortex are highly dynamic, showing rapid morphological changes including elongation/retraction and formation/elimination in response to altered sensory input or neuronal activity, which achieves experience/activity-dependent cortical circuit rewiring. Our previous long-term *in vivo *two-photon imaging study revealed that spine turnover in the mouse primary somatosensory (S1) cortex markedly increased in an early development phase of neuropathic pain, but was restored in a late maintenance phase of neuropathic pain. However, it remains unknown how spine morphology is altered preceding turnover change and whether gain and loss of presynaptic boutons are changed during neuropathic pain.

**Findings:**

Here we used short-term (2-hour) and long-term (2-week) time-lapse *in vivo *two-photon imaging of individual spines and boutons in the S1 cortical layer 1 of the transgenic mice expressing GFP in pyramidal neurons following partial sciatic nerve ligation (PSL). We found in the short-term imaging that spine motility (Δ length per 30 min) significantly increased in the development phase of neuropathic pain, but returned to the baseline in the maintenance phase. Moreover, the proportion of immature (thin) and mature (mushroom) spines increased and decreased, respectively, only in the development phase. Long-term imaging data showed that formation and elimination of boutons moderately increased and decreased, respectively, during the first 3 days following PSL and was subsequently restored.

**Conclusions:**

Our results indicate that the S1 synaptic structures are rapidly destabilized and rearranged following PSL and subsequently stabilized in the maintenance phase of neuropathic pain, suggesting a novel therapeutic target in intractable chronic pain.

## Findings

Neuropathic pain, the effective treatment of which is still lacking, is caused by a lesion along the somatosensory system and lasts for prolonged periods once it developed. Earlier findings from macroscopic brain imaging studies have suggested that maladaptive plastic changes, such as hyperexcitability and reorganization, in the primary somatosensory (S1) cortex play active roles in the chronification of neuropathic pain [1,2]. Recently, we further proposed at the synaptic level the rapid and phase-specific remodeling of neuronal connections in the S1 cortex during neuropathic pain [3], because turnover of dendritic spines in the S1 cortex of living mice markedly increased during the early development phase of neuropathic pain and was restored during the subsequent maintenance phase of neuropathic pain. However, it is still unknown how spine is morphologically changed preceding the occurrence of gain and loss in the differential phases of neuropathic pain. Do presyaptic axonal boutons change their morphology and turnover rate correlated with dendritic spine remodeling during neuropathic pain development? To address these questions, we conducted a short- and long-term *in vivo *two-photon imaging of layer 1 spines and boutons in the S1 cortex of M-line mice, which express GFP in a small subset of layer 5 pyramidal neurons [4], before and after partial sciatic nerve ligation (PSL) [5]. Layer 5 pyramidal neurons are the major output cells in the S1cortex and their distal tuft dendrites in layer 1 that are innervated by thalamocortical and corticocortical long-range projections as well as local circuit inputs, encode information about hind limb stimuli [6].

**Figure 1:**
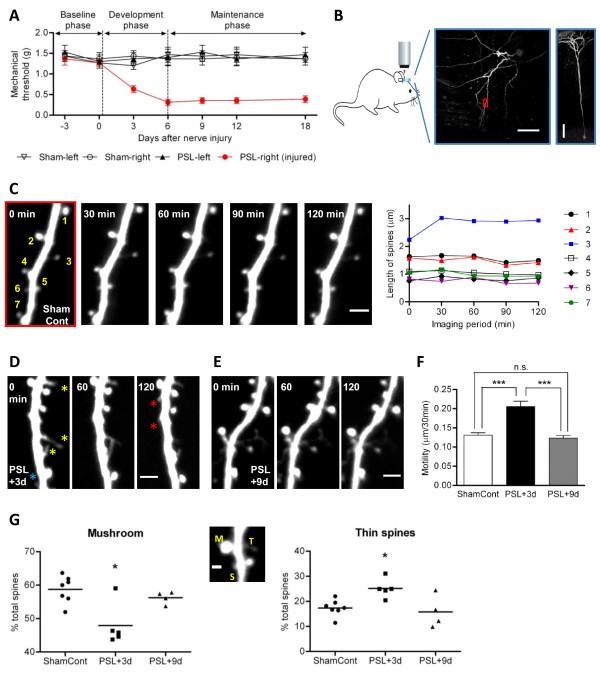
**Changes in spine morphology during peripheral neuropathic pain**. (A) Development of tactile allodynia (~6 d) and its prolonged maintenance (6 d~) in the injured paw of PSL mice (n = 6). Sham, sham-operated mice (n = 6). (B) Left, schematic of *in vivo *imaging. Middle, low-magnification z-projection image of the S1 cortex layer 5 pyramidal cell (scale bar, 50 μm) in a sham control mouse. Right, lateral view of the same cell (scale bar, 100 μm). (C) Left, high-resolution time-lapse image of the same dendritic segment shown in B (red box). Scale bar, 3 μm. Right, lengths of the seven spines numbered in the left panel. (D) Representative *in vivo *time-lapse image of the dendrite taken at PSL+3 d. Note that highly motile thin spines (yellow asterisks) could be seen with high proportion. In addition, a subset of spines exhibited elimination (blue asterisk) and generation (red asterisks) during the 2-h imaging period. (E) Time-lapse image of the dendrite taken at PSL+9 d. (F) Spine motility significantly increased at PSL+3 d (n = 212 spines, 5 mice) and then returned to control (n = 355 spines, 7 mice) level at PSL+9 d (n = 172 spines, 4 mice). ***P < 0.001, one-way ANOVA followed by a Dunnet's multiple comparison test. (G) Proportion of the mushroom (left panel) and thin (right panel) spines significantly decreased and increased, respectively, at PSL+3 d. Such change in spine types was restored at PSL+9 d. *P < 0.05 vs ShamCont or PSL+9 d, one-way ANOVA followed by a Dunnet's multiple comparison test. Middle, representative image showing different types of spines: mushroom (M), stubby (S) and thin (T) spines. Stubby spines were excluded in analysis because they might be in the middle of elimination or formation. Scale bar, 1 μm.

PSL injury in male mice (3-month old) markedly increased mechanical sensitivity of the injured paw with peaking on day 6 and persisting for prolonged periods (*P *< 0.01, repeated measures two-way ANOVA; Figure 1A), indicating that neuropathic pain can be differentiated into the early 'development' (~6 d) and the later 'maintenance' phases (6 d~) [3]. In the short-term time-lapse (30-min intervals for 2 hours) imaging experiments, we first examined a morphological dynamics of spines (i.e. motility: length change per 30-min) in the development phase (PSL+3 d), maintenance phase (PSL+9 d) or control conditions (Figure 1B-F). Spine motility is changed by altered synaptic activity or experience and precedes spine elimination or stabilization [7,8]. In the adult control mice, most spines showed little change in length over the imaging period (Figure 1C), resulting in very low motility (Figure 1F), in line with previous studies using even young mice [9]. Following PSL injury, however, spine motility significantly increased in the development phase (Figure 1D and 1F) and such increase returned to the baseline level in the maintenance phase (Figure 1E and 1F). Since immature new spines are typically thin and motile, and they are subsequently stabilized to the mushroom-type or retracted in an activity/experience-dependent manner [7,10], we further compared the proportion of thin and mushroom spines between the control conditions and differential phases of neuropathic pain. As shown in Figure 1D and 1G, the proportion of thin spines significantly increased whereas that of large mushroom spines was reduced in the development phase of neuropathic pain. Notably, such dramatic transition in spine morphology, together with our previous findings of increased spine density in the development phase [3], resembles the synaptic potentiation-induced dendritic morphogenesis in hippocampal slice preparation [11,12]. Again, the changes in the proportion of spine types were completely restored in the maintenance phase of neuropathic pain (Figure 1G). Re-analysis of our previous long-term imaging data [3] also showed the similar results (data not shown), corroborating the present findings.

**Figure 2:**
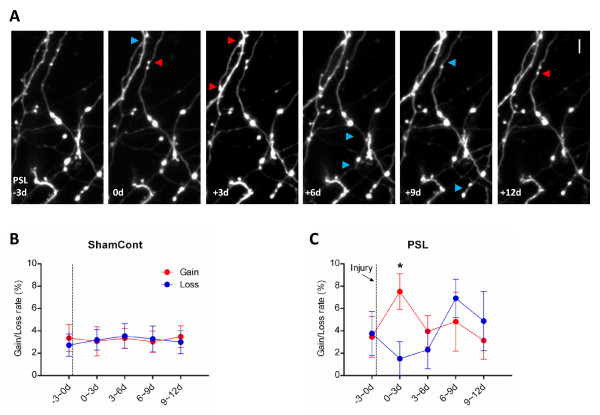
**Changes in formation and elimination of axonal boutons during neuropathic pain**. (A) Long-term time-lapse image of the same axonal segments before and after PSL injury. Arrowheads indicate boutons generated (red) or eliminated (blue) when compared with the previous imaging session. Scale bar, 5 μm. (B) Gain and loss rates of the sham control mice show little change over the 2-week imaging period. (C) Gain and loss rates of the neuropathic mice show moderate increase and decrease only during the first 3 days after PSL (*P < 0.05 vs. loss, paired t-test). Sham control: n = 10 mice (367 boutons); PSL: n = 6 mice (167 boutons).

There was little change in axonal boutons during the short (2-hour) imaging period in any groups of mice, reflecting that boutons are more stable than spines as suggested in earlier studies [13,14]. In the long-term time-lapse (3-day intervals for 2 weeks) imaging experiments, we found that the gain and loss rates of presynaptic boutons moderately increased and decreased during the first 3 days following PSL injury, respectively, and subsequently returned to the baseline level (Figure 2). It should be noted that the density of dendritic spines increased at the development phase and subsequently decreased to control level in the maintenance phase, because spine gain showed a striking increase only during the development phase while spine loss rate increased to a lesser degree for more prolonged period [3]. How could these separate turnover data of boutons and spines be linked? Two different processes of synapse turnover have been suggested: i) a new spine (about 1/3 of total new spines) make a synapse on a new bouton (single-synapse bouton); ii) a new spine (about 2/3 of total new spines) synapse onto an existing bouton that already connected to another spine (transient multi-synapse bouton) and competitive elimination of unnecessary spines subsequently occur [7,15]. Since the increased generation of new boutons and enhanced survival of existing boutons were transiently observed in the early development phase of neuropathic pain and were subsequently restored in the maintenance phase (Figure 2C), the suggested two processes of synapse turnover might occur simultaneously following neuropathic injury. In addition to the bouton turnover, we analyzed the volume change of boutons before and after PSL injury by measuring the normalized brightness of each boutons. Although there seemed to be a slight increase in bouton volume following injury (normalized brightness: 1.01 ± 0.06 at PSL-3 d; 1.09 ± 0.08 at PSL+3 d; 1.06 ± 0.07 at PSL+6 d; 1.07 ± 0.08 at PSL+9 d; 1.03 ± 0.06 at PSL+12 d, n = 26), no significant change was observed (*P >*0.9, one-way ANOVA).

In summary, spine morphology and bouton turnover in the S1 cortex are phase-specifically changed following PSL injury, correlating with long-term spine remodeling data in our previous study [3]. Thus, it can be collectively suggested that synaptic structures in the S1 cortex are destabilized and rearranged in the early development phase of neuropathic pain and subsequently stabilized in the late maintenance phase. Such stabilization of new circuits might be a structural correlate of long-lasting nature of neuropathic pain, as represented by the sustained head enlargement of new persistent spines that were generated in the development phase during the late phase of neuropathic pain [3]. Investigations to clarify whether and how the S1 synapse remodeling directly contribute to neuropathic pain behaviors are currently in progress.

## Materials and methods

All animal experiments were approved by the Animal Research Committee of the National Institutes of Natural Sciences. For the short-term imaging, GFP-M-line mice (male, 3-month old) were implanted with a small open-skull glass window over the left S1 cortex under urethane anesthesia (1.9 g/kg) as described previously [16]. High-resolution imaging of dendrites (0.08 μm/pixel, 15-30 optical planes, 0.5 μm z-step) or axons (0.14 μm/pixel, 10-30 optical planes, 1.0 μm z-step) within 100 μm below the cortical surface (i.e. layer 1) were performed every 30 min for 2 hours with two-photon laser scanning microscopy (Olympus FV1000MPE microscope, Spectra-Physics Mai Tai Ti:sapphire laser at 950 nm). The location of imaged dendrites/axons was then confirmed inside the S1 hind paw area by using intrinsic optical signal imaging [17]. For the long-term imaging, 2-month old M-line mice were implanted with a glass window and repeated two-photon imaging of the same axons (3-day intervals for 2 weeks) within the S1 hind paw area under isoflurane anesthesia (~1.5%) was started at 5 weeks after the window implantation [3]. Metamorph (Molecular Devices) and ImageJ (http://rsbweb.nih.gov/ij/) were used to analyze individual spines and boutons from three-dimensional image stacks, blind to the experimental conditions. Detailed criteria for scoring, classifying and analyzing the spines and boutons are described elsewhere [3,9,16,18]. Briefly, only spines that were clearly protruded from the shaft (> 0.4 μm laterally) and boutons that were three times brighter than the shaft were included in analysis. Spine length was measured from the dendritic shaft to the tip of spines and the motility was calculated as average absolute Δ length per 30 min. Spines were classified into three different types: mushroom (the diameter of spine head is two times larger than neck diameter and the length of spine head is longer than neck length), stubby (no head & total spine length is less than 1 μm) and thin (the length of spine head is shorter than neck length & total spine length is longer than 1 μm). Changes in the gain and loss rates of boutons were determined as the percentages of bouton formation and elimination between two successive imaging sessions, relative to the total bouton number in the former session. Only boutons that clearly separated from other bouton-like swellings over the 2-week imaging period were included in the analysis of integrated brightness, which was calculated by summing the intensity of all pixels comprising a bouton after background subtraction, divided by mean intensity of the adjacent axonal shaft. Each integrated brightness value was normalized to the value at PSL 0 d. For PSL injury, the right sciatic nerve was exposed at high-thigh level and 1/3-1/2 the diameter of the nerve was ligated with 9-0 suture. For sham-operation, the nerve was exposed, but left intact. The behavioral sign of tactile allodynia was assessed by using von Frey hair test. Data are presented as mean ± s.e.m. *P *values were calculated using a paired t-test or one-way ANOVA followed by a Dunnet's multiple comparison test unless otherwise stated.

## Competing interests

The authors declare that they have no competing interests.

## Authors' contributions

SKK, GK and JN designed the study and wrote the manuscript. SKK and TI performed the experiments and analysis. All authors read and approved the final manuscript.

## References

[B1] Costigan M Scholz J Woolf CJ Neuropathic pain: A maladadaptive response of the nervous system to damage Annu Rev Neurosci 2009 32 1 32 10.1146/annurev.neuro.051508.135531 19400724 PMC2768555

[B2] Seifert F Maihofner C Central mechanisms of experimental and chronic neuropathic pain: findings from functional imaging studies Cell Mol Life Sci 2009 66 375 390 10.1007/s00018-008-8428-0 18791842 PMC11131450

[B3] Kim SK Nabekura J Rapid synaptic remodelling in the adult somatosensory cortex following peripheral nerve injury and its association with neuropathic pain J Neurosci 2011 31 5477 5482 10.1523/JNEUROSCI.0328-11.2011 21471384 PMC6622722

[B4] Feng G Mellor RH Bernstein M Keller-Peck C Nguyen QT Wallace M Nerbonne JM Lichtman JW Sanes JR Imaging neuronal subsets in transgenic mice expressing multiple spectral variants of GFP Neuron 2000 28 41 51 10.1016/S0896-6273(00)00084-2 11086982

[B5] Malmberg AB Basbaum AI Partial sciatic nerve injury in the mouse as a model of neuropathic pain: behavioral and neuroanatomical correlates Pain 1998 76 215 222 10.1016/S0304-3959(98)00045-1 9696476

[B6] Murayama M Perez-Garci E Nevian T Bock T Senn W Larkum ME Dendritic encoding of sensory stimuli controlled by deep cortical interneurons Nature 2009 457 1137 1141 10.1038/nature07663 19151696

[B7] Holtmaat A Svoboda K Experience-dependent structural synaptic plasticity in the mammalian brain Nat Rev Neurosci 2009 10 647 658 10.1038/nrn2699 19693029

[B8] Harms KJ Dunaevsky A Dendritic spine plasticity: looking beyond development Brain Res 2007 1184 65 71 16600191 10.1016/j.brainres.2006.02.094

[B9] Tropea D Majewska AK Garcia R Sur M Structural dynamics of synapses in vivo correlate with functional changes during experience-dependent plasticity in visual cortex J Neurosci 2010 30 11086 11095 10.1523/JNEUROSCI.1661-10.2010 20720116 PMC2932955

[B10] Yasumatsu N Matsuzaki M Miyazaki T Noguchi J Kasai H Principles of long-term dynamics of dendritic spines J Neurosci 2008 28 13592 13608 10.1523/JNEUROSCI.0603-08.2008 19074033 PMC2706274

[B11] Engert F Bonhoeffer T Dendritic spine changes associated with hippocampal long-term synaptic plasticity Nature 1999 399 66 70 10.1038/19978 10331391

[B12] Maletic-Savatic M Malinow R Svoboda K Rapid dendritic morphogenesis in CA1 hippocampal dendrites induced by synaptic activity Science 1999 283 1923 1927 10.1126/science.283.5409.1923 10082466

[B13] Deng J Dunaevsky A Dynamics of dendritic spines and their afferent terminals: spines are more motile than presynaptic boutons Dev Biol 2005 277 366 377 10.1016/j.ydbio.2004.09.028 15617680

[B14] Majewska AK Newton JR Sur M Remodeling of synaptic structure in sensory cortical areas in vivo J Neurosci 2006 26 3021 3029 10.1523/JNEUROSCI.4454-05.2006 16540580 PMC6673961

[B15] Knott GW Holtmaat A Wilbrecht L Welker E Svoboda K Spine growth precedes synapse formation in the adult neocortex in vivo Nat Neurosci 2006 9 1117 1124 10.1038/nn1747 16892056

[B16] Holtmaat A Bonhoeffer T Chow DK Chuckowree J De Paola V Hofer SB Hübener M Keck T Knott G Lee WA Mostany R Mrsic-Flogel TD Nedivi E Portera-Cailliau C Svoboda K Trachtenberg JT Wilbrecht L Long-term, high-resolution imaging in the mouse neocortex through a chronic cranial window Nat Protoc 2009 4 1128 1144 10.1038/nprot.2009.89 19617885 PMC3072839

[B17] Eto K Wake H Watanabe M Ishibashi H Noda M Yanagawa Y Nabekura J Inter-regional Contribution of Enhanced Activity of the Primary Somatosensory Cortex to the Anterior Cingulate Cortex Accelerates Chronic Pain Behavior J Neurosci 2011 31 7631 7636 10.1523/JNEUROSCI.0946-11.2011 21613476 PMC6633125

[B18] Takatsuru Y Fukumoto M Yoshitomo M Nemoto T Tsukada H Nabekura J Neuronal circuit remodeling in the contralateral cortical hemisphere during functional recovery from cerebral infarction J Neurosci 2009 29 10081 10086 10.1523/JNEUROSCI.1638-09.2009 19675241 PMC6664978

